# Individual and Environmental Factors Influencing Influenza Transmission: A Multilevel Analysis

**DOI:** 10.1111/irv.70232

**Published:** 2026-02-04

**Authors:** Nushrat Nazia, Eleanor Pullenayegum, Mark Loeb

**Affiliations:** ^1^ Department of Pathology & Molecular Medicine McMaster University Hamilton Ontario Canada; ^2^ Child Health Evaluative Sciences, The Hospital for Sick Children Toronto Ontario Canada; ^3^ Dalla Lana School of Public Health The University of Toronto Toronto Ontario Canada

**Keywords:** Bayesian hierarchical model, Canada, environmental exposure, epidemiology, human, influenza, respiratory tract infections, vaccination

## Abstract

**Background:**

Influenza transmission is influenced by both individual characteristics and community‐level drivers. Understanding how these drivers jointly influence transmission is important to predicting outbreaks and guiding influenza prevention strategies. Our study aimed to assess individual and colony‐level influences, including vaccination and environmental factors, on influenza transmission in the Hutterite communities.

**Methods:**

We analyzed data from 3271 individuals in 46 Canadian Hutterite colonies during the 2008 influenza season. Weekly PCR‐confirmed Influenza A and B outcomes were examined in relation to demographic, vaccination, geographic, and weather variables using multilevel Bayesian hierarchical models in Integrated Nested Laplace Approximations (INLA), which accounted for colony clustering and temporal autocorrelation.

**Results:**

Of the 3271 participants, 239 (7.3%) had PCR‐confirmed influenza (128 Influenza A and 111 Influenza B cases). Older age was found to be protective, especially for Influenza B, while males had slightly lower odds than females. Individual vaccination showed little effect, while colony assignment to influenza vaccination was associated with a lower risk of Influenza A and overall Influenza (A/B). Higher weekly mean temperatures were associated with lower odds of Influenza A but with higher odds of Influenza B. Precipitation showed weak associations, and geographic factors such as elevation and distance to the nearest city suggested possible protective effects, but results were imprecise.

**Conclusions:**

Our findings suggest that influenza risk in Hutterite colonies is associated with local environmental and geographic characteristics in addition to the individual drivers. Incorporating the community‐level environmental setting in influenza surveillance may improve preparedness for future outbreaks.

## 1 Introduction

Influenza, an acute respiratory illness, is a major cause of morbidity and mortality worldwide and can spread rapidly in closed or semiclosed communities [1]. Seasonal influenza, common in all parts of the world, affects about a billion people annually, causing 3 to 5 million cases of severe illness and an estimated 290,000 to 650,000 respiratory deaths [2]. Vaccination is well recognized to reduce the risk of influenza [3, 4, 5]. Nevertheless, influenza continues to be a major public health threat despite decades of global surveillance efforts, pharmaceutical, and nonpharmaceutical interventions [6, 7, 8]. In addition to the direct health impacts, influenza can cause a heavy burden on healthcare systems through sudden increases in outpatient visits and hospital admissions, leading to an increased resource demand [9].

Influenza is highly infectious, and transmission is easily spread through individual‐level factors such as age, underlying health conditions, and vaccination status [10, 11, 12]. Community‐level environmental drivers, including temperature, precipitation, geography, and social structure, may also affect spread. Although geographical and environmental conditions have been shown to influence the timing and the intensity of epidemics, uncertainty remains regarding community‐level environmental factors [13, 14, 15]. Traditional epidemiological study designs, such as ecological or individual‐level studies, do not fully account for this hierarchical structure, where individuals are nested within communities [16]. However, in many real‐world settings, this type of hierarchy is difficult to define because communities are often porous and individuals belong to multiple overlapping groups, such as households, schools, or workplaces. In contrast, the Hutterite colonies provide a setting where each individual belongs to one well‐defined and socially cohesive community, which allows the hierarchy to be clearly represented in the analysis. Analytical approaches that explicitly model this hierarchy, such as multilevel or hierarchical models, can provide more precise and reliable inferences about how both community and individual‐level factors shape infection risk [17, 18, 19].

Therefore, the Hutterite colonies in the Canadian prairies provide a unique opportunity to study influenza transmission because of their close‐knit community structure, where members share a similar lifestyle, activities, and population structures with limited external contact [20]. Earlier studies in this community reported that, despite these similarities, some colonies experienced outbreaks while others had none [20, 21].This highlights the variations across colonies despite the similarities in population settings, most likely due to the colony‐level variations.

Our study aimed to identify the individual, community, and environmental determinants of influenza infection in Hutterite colonies using a multilevel modeling framework. Specifically, we examined whether weekly changes in temperature and precipitation were associated with influenza transmission, while adjusting for individual characteristics, vaccination status, and colony‐level features.

## 2 Methods

### 2.1 Study Design, Population, and Settings

**FIGURE 1:**
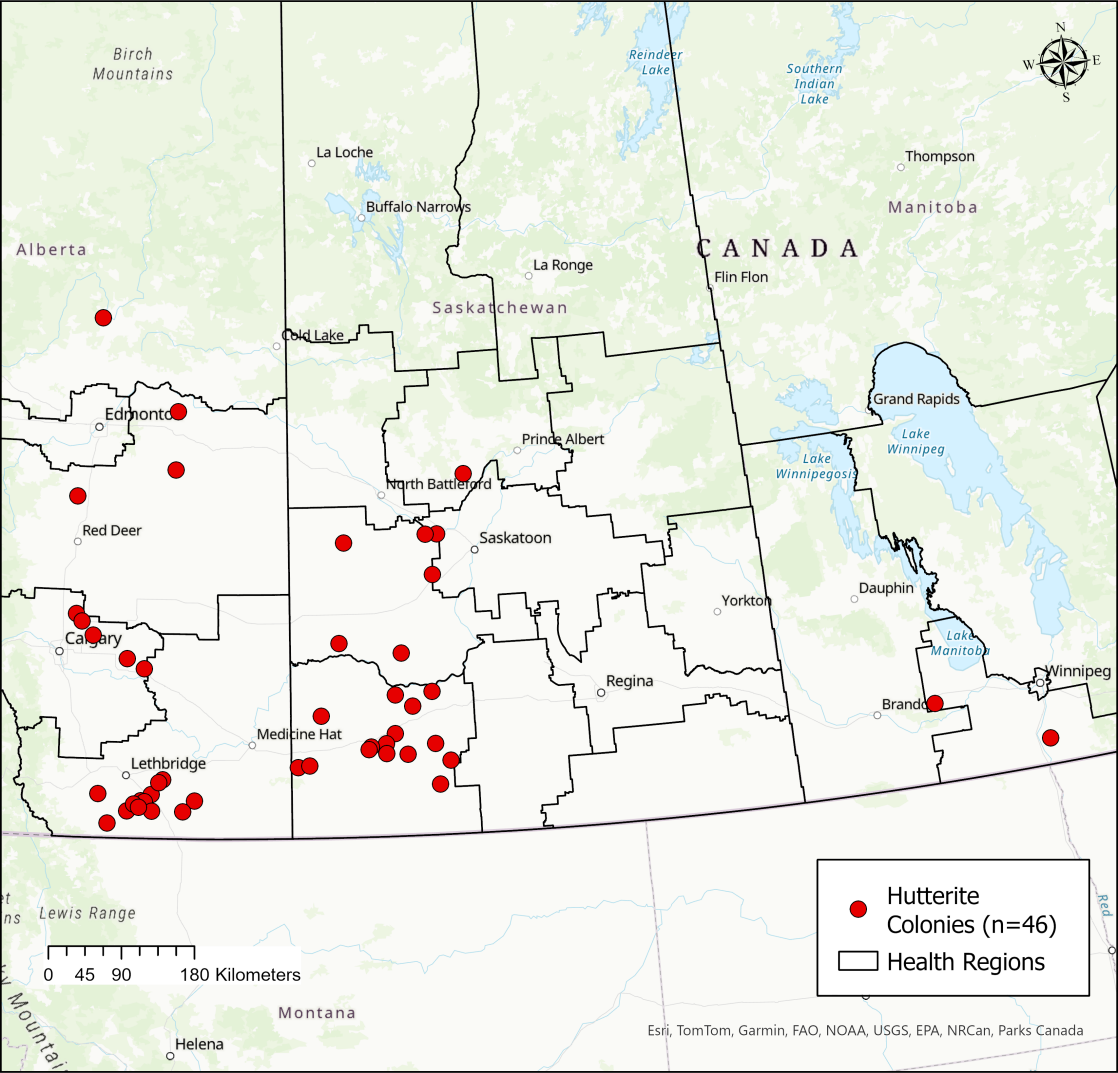
Location of the 46 Hutterite colonies included in the study across Alberta, Saskatchewan, and Manitoba. Hutterite colony points represent approximate locations based on aggregated study data. Maps were generated in ArcGIS Pro (Esri, Redlands, CA, USA) using provincial boundary shapefiles from the Government of Canada Open Data portal.

Our study used data from a prospective cohort established within a cluster‐randomized trial conducted during the 2008 influenza season. Participants were recruited from 46 Hutterite colonies across eight health regions in Alberta, Saskatchewan, and Manitoba who met the study eligibility criteria and provided consent to participate [20]. In the original trial, colonies were randomized to vaccination of children aged 3 years to 15 years with either the inactivated influenza vaccine or the Hepatitis A vaccine as a control group [20]. For the present analysis, we included all individuals residing in participating colonies, regardless of age or vaccination assignment, to examine individual and community‐level determinants of influenza infection. All residents were eligible for weekly surveillance. Figure 1 shows the geographic distribution of these colonies across Canada. The research protocol was approved by the McMaster University Research Ethics Review Board.

### 2.2 Data and Sources

The primary outcomes were laboratory‐confirmed influenza infections, categorized in three ways: any Influenza (A or B), Influenza A only, and Influenza B only. Weekly active surveillance was conducted in all colonies, and respiratory specimens were collected from participants who developed influenza‐like illness symptoms. Influenza infection was confirmed by real‐time reverse transcriptase polymerase chain reaction (RT‐PCR) [20]. Although the randomized trial primarily evaluated the indirect protection of vaccinating children [20], our analysis included all participating individuals in the 46 colonies, regardless of their age or vaccination status, to assess individual, environmental, and community‐level factors across all participants. For each participant, demographic information (age and sex) and clinical details, such as influenza vaccination status and presence of high‐risk conditions, were collected from the baseline dataset during the 2008–2009 influenza season (December 22, 2008, to June 28, 2009). In addition, colony‐level study group assignment (influenza vaccine vs. Hepatitis A vaccine) was included to account for the cluster‐randomized design of the original trial [20].

Colony‐level geographic and environmental datasets included elevation, distance to the nearest city, and weekly weather data. Elevation data for each colony were obtained from the Canadian Digital Elevation Model available through the Government of Canada's Open Data portal (https://open.canada.ca/data/en/dataset/18752265‐bda3‐498c‐a4ba‐9dfe68cb98da) and processed using ArcGIS Pro 3.1.0 software. Distances from each colony to the nearest city were calculated in ArcGIS Pro 3.1.0 using geodesic measurements between colony centroids and the nearest urban center. Daily weather data were obtained from Environment and Climate Change Canada's Historical Climate Data portal (https://climate.weather.gc.ca) from the nearest meteorological station to each colony. Colony locations were linked to their corresponding weather station. Daily temperature and precipitation were aggregated to the weekly averages for analysis.

### 2.3 Statistical Analysis

We used a generalized linear mixed model (GLMM) with a logistic link to evaluate influenza infection risk, accounting for hierarchical clustering at the colony level and temporal autocorrelation across weeks [22]. Three outcomes were analyzed separately: any influenza (Influenza A or B), Influenza A, and Influenza B. Models were estimated in R‐INLA (R Studio 2024.04.2), which uses Integrated Nested Laplace Approximation (INLA) [23, 24], providing efficient Bayesian inference for latent Gaussian models by approximating the posterior distributions.

The binary outcome variable $y_{\mathrm{ijt}}$ represents whether the individual i in colony j had PCR‐confirmed influenza infection (1, *Yes*; 0, *No*) during week t. We modeled the log odds of infection of influenza as

(1) $$\begin{array}{c} \text{logit}\left(P\left(y_{\mathrm{ijt}}=1\right)\right)=\beta_{0}+\beta_{1}\;{\mathrm{Sex}}_{\mathrm{ij}}+\beta_{2}\;{\mathrm{Age}}_{\mathrm{ij}}+\beta_{3}\;{\text{HighRisk}}_{\mathrm{ij}}+\beta_{4}\;{\text{Vaccinated}}_{\mathrm{ij}}\\ \;+\beta_{5}\text{Study}{\text{Group}}_{\mathrm{ij}}+\beta_{6}\;\;{\text{Elevation}}_{\mathrm{ij}}+\beta_{7}\;\;\text{City}\_{\text{Dist}}_{\mathrm{ij}}\\ \;+\beta_{8}\;\;{\text{Temp}}_{\mathrm{jt}}+\beta_{9}\;{\text{Prec}}_{\mathrm{jt}}+u_{j}+w_{t} \end{array}$$

*where i* indexes the individual, *j* indexes the colony, and *t* indexes the week.

- Individual‐level covariates: sex (male = 0, female = 1), age (continuous), vaccination status (0 = vaccinated, 1 = not vaccinated within or outside the trial), and presence of high‐risk conditions (0 = no, 1 = yes)
- Colony‐level covariates: study group assignment (1 = influenza intervention, 0 = hep B/placebo), elevation (meters), and distance to the nearest city (kilometers).
- Time‐varying cluster‐level covariates: ${\text{Temp}}_{\mathrm{jt}}$ is the mean temperature for colony *j* at week *t,* and ${\text{Prec}}_{\mathrm{jt}}$ is the mean total precipitation for colony *j* at week *t*.

The colony‐level random effect was specified as

(2) $$u_{j}\sim N\left(0,\sigma_{u}^{2}\right)$$

We included a weekly random effect $w_{t}$ modeled as an AR (1) process:

(3) $$w_{t}=\rho w_{t-1}+\delta_{t},\delta_{t}\sim N\left(0,\sigma_{w}^{2}\;\right),\;\left|\rho \right|<1$$

where $w_{t}$ is the week‐specific temporal random effect, ρ is the AR (1) serial autocorrelation parameter, [25, 26], which measures how strongly each week's outcome is correlated with the previous week, and $\delta_{t}$ is the random noise. Prior research has also accounted for weekly autocorrelation in influenza models [14, 27].We also evaluated an alternative specification that allowed colony‐specific week effects (random slopes for week within colony) to capture potential differences in temporal influenza dynamics across colonies. However, this structure did not offer a clear improvement in model performance or interpretability. Given the limited number of weekly observations and largely concurrent epidemic activity across colonies, we retained a more parsimonious model with colony‐level random intercepts and a single autoregressive (AR1) week effect common to all colonies. Each participant experienced only one PCR‐confirmed influenza episode during the study and therefore, individual‐level random intercepts were not included in the model.

We used penalized complexity (PC) priors developed by Simpson et al. for the colony‐level and temporal random effects [28, 29]. These priors shrink towards a simpler model with no random effect but allow variability and are defined through probability statements on the standard deviation [29, 30]. In comparison to the vague priors, the pc priors also tend to avoid overfitting [14, 28, 31]. In our models, we set the prior so that the probability of the random‐effect standard deviation exceeding 0.5 was 0.01 ($P\left(\sigma >0.5\right)=0.01$).

Overall, we ran seven different model versions for each of the three influenza outcomes considered in this study. The models varied by whether weather variables were used as continuous or categorical (0‐ or 1‐week lag), inclusion of vaccination fixed‐effects terms, and whether the previous week's influenza incidence was considered.

### 2.4 Model Fit and Diagnostics

The model fit was evaluated using the Deviance Information Criterion (DIC), Watanabe–Akaike Information Criterion (WAIC), and log pseudomarginal likelihood (LPML). Among all model formulations (Models 1–6), these indicators were broadly similar, suggesting comparable fit with varying weather specifications and vaccination covariates. The 1‐week lagged model (Model 5) was selected as the final specification because it aligned with the biological timing of influenza transmission and showed interpretable, linear relationships between temperature, precipitation, and infection risk (Appendix B in the Supporting Information). The random‐effect hyperparameters indicated meaningful colony‐level variation and moderate week‐to‐week autocorrelation. A post hoc model including previous week colony‐level influenza activity and additional sensitivity analyses (Appendix A in the Supporting Information) produced consistent findings.

## 3 Result

### 3.1 Descriptive Statistics

Table 1 summarizes the descriptive characteristics of the study dataset (46 colonies with 3271 participants). The mean participant age was 26 years, with 53.3% aged 16–64 years, 36.2% aged 3–15 years, 5.6% under 2 years, and 4.9% aged ≥ 65 years. 43.5% were female, and 24.3 were vaccinated against influenza either within or outside the trial. Twenty‐two colonies (1498 participants) were in the influenza vaccine study group, and 24 (1773 participants) colonies were in the Hepatitis A vaccine control study group. Overall, 239 participants had RT‐PCR‐confirmed influenza, including 128 Influenza A and 111 Influenza B positive cases. Environmental characteristics included a mean colony elevation of 801.2 m and the mean distance of 15.6 km to the nearest city. The average weekly temperature across the 46 colonies was 0.9°C (SD 10.0), and mean weekly precipitation was 0.8 mm (SD 1.7).

**TABLE 1:** Summary statistics.

Variable	Value
Individual characteristics (*n* = 3271)	
Age, mean (SD), *y*	26.0 (20)
Age groups, *n* (%)	
—0–2 years	184 (5.6%)
—3–15 years	1183 (36.2%)
—16–64 years	1744 (53.3%)
—≥ 65 years	160 (4.9%)
Female sex, *n* (%)	1423 (43.5%)
Vaccinated against influenza, *n* (%)	796 (24.3%)
Study group characteristics	
Colonies randomized to the influenza vaccine	22 colonies,1498 participants
Colonies randomized to Hepatitis A vaccine (control)	24 colonies, 1773 participants
Influenza outcomes	
RT‐PCR‐confirmed Influenza (any A or B)	239 (46.5%)
—Influenza A only, *n* (%)	128 (53.5%)
—Influenza B only, *n* (%)	111 (3.4%)
Participants with > 1 flu episode, *n* (%)	0 (0.0%)
Total observation weeks (Season 1)	22
Cluster/colony characteristics	
Number of colonies	46
Mean participants per colony (SD)	71.1 (25.3)
Mean vaccinated per colony (SD)	17.3 (14.4)
Environmental characteristics	
Elevation, mean (m)	801.2
Distance to nearest city, mean (km)	15.6.
Mean temperature (°C), mean (SD)	0.9 (10.0)
Total precipitation (mm), mean (SD)	0.8 (1.7)

Values are summarized for 3271 participants across 46 colonies during the 2008 influenza season.

RT‐PCR = reverse transcriptase polymerase chain reaction; SD = standard deviation.

**FIGURE 2:**
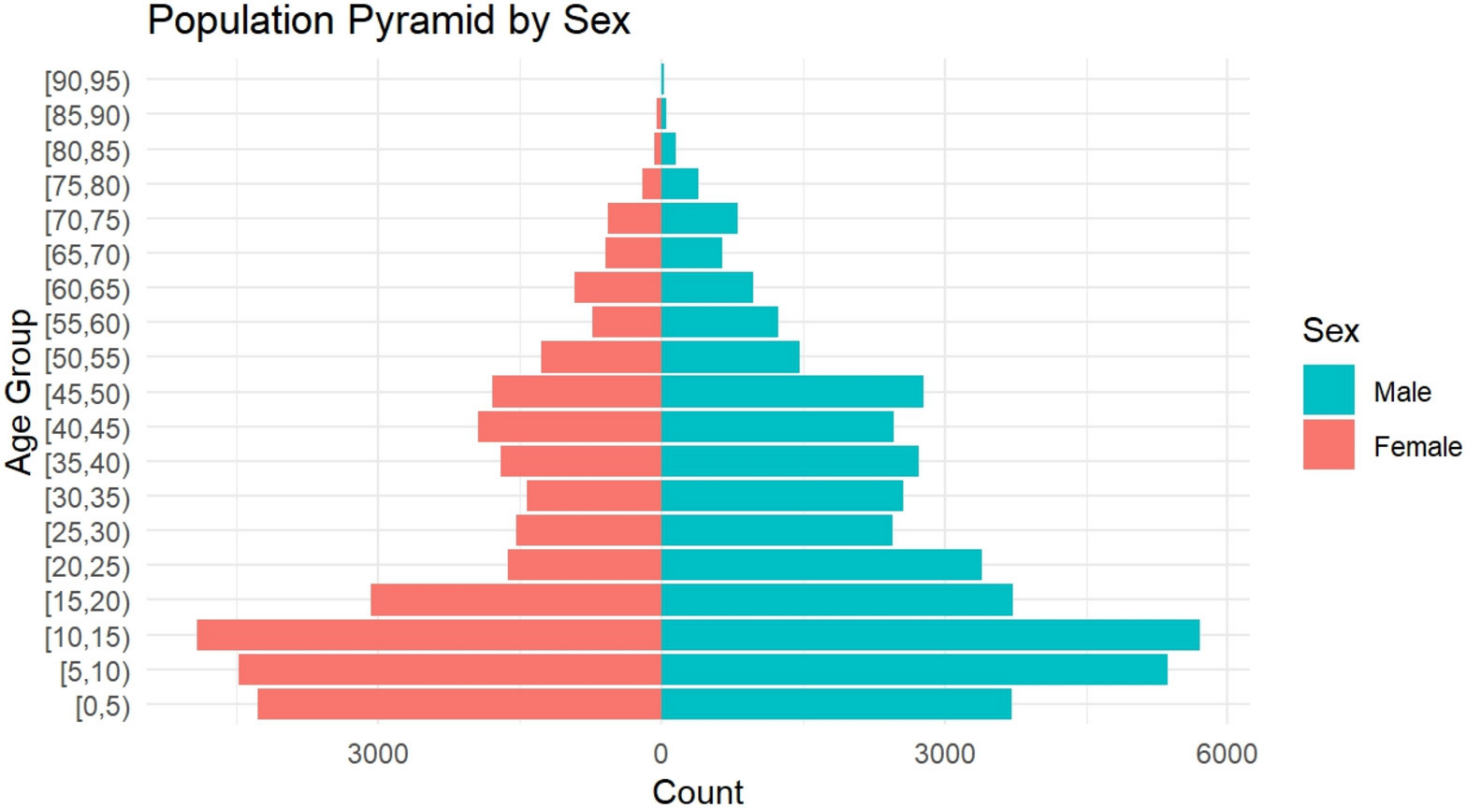
Population pyramid by sex showing the age and sex distribution of participants across all colonies.

**FIGURE 3:**
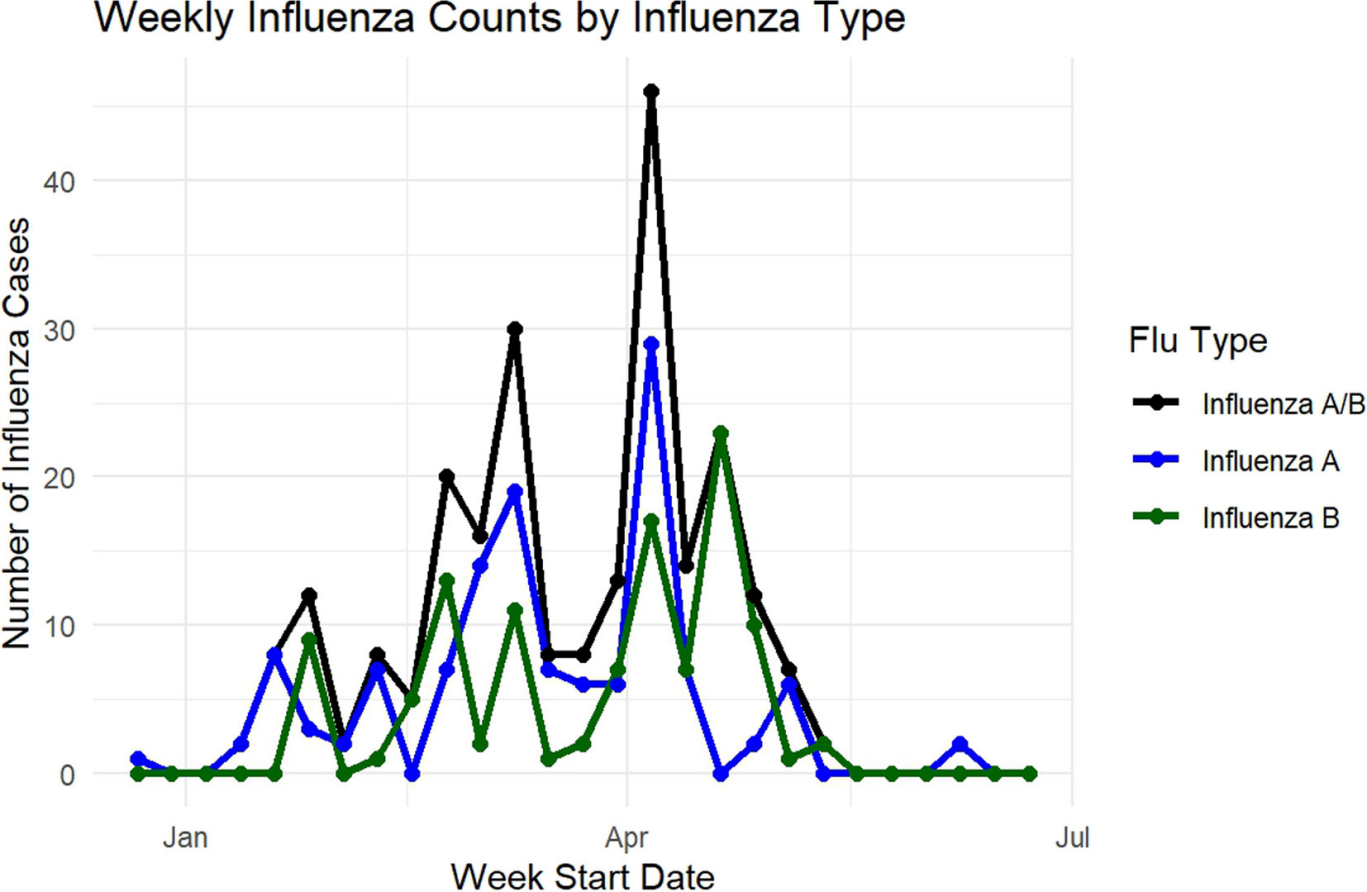
Weekly influenza counts by virus type (A and B) across all colonies during the 2008 influenza season.

Figure 1 shows the population pyramid by sex of the study participants, highlighting the relatively young age structure of the population with larger numbers of children and adolescents, compared with older age groups. Figure 2 shows the temporal distribution of influenza, with peaks for both Influenza A and B between February and April. The highest counts were observed in early April. Figure 3 represents the weekly influenza counts by virus type across all colonies during the 2008 influenza season.

### 3.2 Multilevel Model Findings

The 1‐week lagged model was retained as the final specification because it aligned with the biological timing of influenza transmission and provided comparable fit across alternative model formulations. Sensitivity analyses using alternative lag structures, categorical weather terms, and exclusion of zero‐case colonies produced consistent results Appendix A in the Supporting Information.

Table 2 shows the fixed effect estimates from the final Bayesian hierarchical model for the three outcomes: Influenza A/B (all influenza), Influenza A, and Influenza B. Female sex was associated with a lower risk of influenza infection across all outcomes, though the evidence for this association was weak based on credible intervals that include values near 1. Increasing age was consistently found to be protective, with a stronger effect observed for Influenza B (RR = 0.93, 95% CrI: 0.91–0.95) compared to Influenza A (RR = 0.99, 95% CrI: 0.98–1.00). Presence of existing high‐risk health condition showed little evidence of an association with overall influenza (RR = 0.95, 95% CrI: 0.67–1.33) but suggested increased risk for Influenza A (RR = 1.29, 95% CrI: 0.83–2.00) and reduced risk for Influenza B (RR = 0.41, 95% CrI: 0.22–0.76). Individual vaccination was not found to be protective in any model outcomes. However, being assigned to the influenza vaccine study group was associated with substantially reduced risk for all influenza (RR = 0.29, 95% CrI: 0.10–0.83) and Influenza A (RR = 0.17, 95% CrI: 0.04–0.62), but not Influenza B. Distance to the nearest city and elevation were weakly associated with reduced risk but with wide uncertainty. Among the two weather variables, higher temperature in the previous week was associated with decreased risk of Influenza A (RR = 0.91, 95% CrI: 0.86–0.95) and increased risk of Influenza B (RR = 1.19, 95% CrI: 1.09–1.31), while precipitation showed a weak positive association across all outcomes.

**TABLE 2:** Fixed effect estimates from the multilevel model for Influenza A/B, Influenza A, and Influenza B outcomes.

Variables	All Influenza (RR, 95% CrI)	Influenza A (RR, 95% CrI)	Influenza B (RR, 95% CrI)
Sex (female vs. male)	0.79 (0.60–1.02)	0.77 (0.54–1.11)	0.75 (0.50–1.10)
Age (per year)	0.97 (0.96–0.98)	0.99 (0.98–1.00)	0.93 (0.91–0.95)
High‐risk status	0.95 (0.67–1.33)	1.29 (0.83–2.00)	0.41 (0.22–0.76)
Individual vaccination	1.24 (0.86–1.79)	0.87 (0.51–1.50)	1.08 (0.57–2.05)
Study group assignment (influenza or placebo)	0.29 (0.10–0.83)	0.17 (0.04–0.62)	0.99 (0.23–4.43)
Distance to the city	0.63 (0.34–1.12)	0.65 (0.31–1.30)	0.70 (0.31–1.53)
Elevation	0.73 (0.45–1.21)	1.04 (0.56–1.97)	0.62 (0.32–1.19)
Temperature (lag‐1 week)	1.00 (0.96–1.05)	0.91 (0.86–0.95)	1.19 (1.09–1.31)
Precipitation (lag‐1 week)	1.07 (0.91–1.27)	1.11 (0.87–1.41)	1.11 (0.88–1.39)

Values are exponentiated posterior means with 95% credible intervals (CrI).

Models included random intercepts for colony (IID) and week (AR1).

## 4 Discussion

Our study investigated the influenza transmission dynamics within 46 Hutterite colonies by assessing individual and colony‐level factors, including environmental and geographic influences, using a multilevel Bayesian hierarchical modeling framework. The findings from our study indicate that older age was associated with a lower risk of infection, particularly for Influenza B, while male participants had a slightly lower risk than female participants.

Colony‐level environmental and geographic factors showed different patterns across the three outcomes: all influenza, Influenza A, and Influenza B. For instance, higher mean temperatures in the previous week were associated with lower odds of Influenza A and higher odds of Influenza B, suggesting distinct ecological influences on each virus type. Prior literature shows that Influenza B often peaks later in the season than Influenza A, which supports the plausibility of subtype‐specific temperature associations in a single‐season analysis [32]. This seasonal offset may contribute to the opposite temperature relationships observed here, as Influenza A and B circulated at different points within the winter season. Because our analysis reflects a single influenza season, these findings should be interpreted cautiously. The observed associations may partly reflect the timing of subtype circulation during 2008–2009 rather than stable mechanistic differences in temperature responsiveness. Further multiseason analyses would be required to determine whether these patterns are consistent across years.

Precipitation showed weaker and less consistent associations with wide uncertainty in the estimates. Colony elevation and distance to the nearest city also suggested some possible associations, but the estimates were imprecise. The use of 1‐week lagged weather terms provided biologically plausible insights consistent with incubation periods and short‐term transmission dynamics. Overall, our findings are in line with prior research suggesting that weather and geography may shape the timing and intensity of influenza outbreaks [15, 33, 34]. However, once transmission is established, person‐to‐person spread likely becomes the primary mechanism sustaining influenza spread within colonies.

Individual‐level vaccination status was not significantly associated with reduced risk in our multilevel models. In this trial, only children received the influenza or Hepatitis A vaccine according to their colony's randomized assignment. Adults did not receive any vaccine as part of the trial, so any adult vaccination status recorded in the dataset is subject to confounding by factors such as age or susceptibility. This may contribute to the weaker individual‐level association. In contrast, the colony‐level vaccination term reflects the effect of the randomized intervention and therefore captures the primary variation in vaccination‐related transmission risk. This should not be interpreted as the absence of direct protection. The strong protective association at the colony level is consistent with indirect (herd) effects in these closely connected communities and aligns with the original trial findings by Loeb et al., which showed that vaccinating children reduced community‐level influenza risk using the same dataset [20]. The effect of vaccinating children against influenza reduced outbreaks in the influenza vaccine‐assigned colonies by about half. This suggests that community‐level vaccine coverage and herd effect are particularly important, and communal, social, and environmental conditions may contribute to shaping an outbreak risk. Our post hoc model included the presence of influenza cases from the past week at the colony level. The findings of the post hoc model are consistent with prior studies that recent local transmission strongly influences ongoing spread [14, 35], providing additional support to the main results. Altogether, these findings indicate that influenza risk in a community setting is determined by both individual‐level vulnerability and community‐level dynamics, with weather acting as a modifying factor. This multilevel perspective emphasizes the need for strategies that combine high vaccine uptake with careful monitoring of environmental and epidemiological indications.

The strength of our study includes the unique Hutterite study setting, which provides a rare opportunity to study influenza transmission in closely monitored and well‐defined communities with limited exposure to the outside. The surveillance and cluster‐randomized trial study design with integration of geographic and environmental data enabled detailed modeling of both individual and colony‐level factors. The use of Bayesian hierarchical models with temporal autocorrelation allowed us to account for the week‐to‐week dependence in influenza activity, providing more reliable inference.

Our study also has some limitations. Weather data were derived from the nearest meteorological stations and averaged to the colony level, which may not fully capture local variations. Only one influenza season was included, limiting the ability to assess year‐to‐year variability. Post hoc analyses, including the incorporation of the previous week's colony‐level activity, were primarily exploratory and should be interpreted with caution. There may also have been other unmeasured factors, such as prior immunity or within‐colony contact patterns, that influenced influenza risk but were not captured in this analysis. Finally, the findings of our study may not be generalizable beyond similar communal populations, where contact patterns and social structures may differ from those of urban or more heterogeneous communities. The Hutterite colonies function as close‐contact communities with relatively stable and uniform contact patterns, which differ substantially from more heterogeneous or urban community settings. This semiclosed colony structure also allows certain transmission mechanisms to be observed with less confounding, as exposure to outside sources is limited. While these features strengthen inference about how community‐level factors, including vaccination coverage and environmental conditions, influence influenza spread, the effect sizes observed here may not translate directly to broader populations. Nonetheless, the mechanisms identified may still be relevant in other communal or institutional settings with similar structured contact patterns.

To summarize, our findings show that influenza transmission in Hutterite colonies is a complex process shaped by both individual characteristics and colony‐level dynamics. These results highlight the importance of community‐level environmental factors in influenza risk. Overall, this study contributes to the understanding of influenza epidemiology by showing how community structure and environmental exposures interact with individual‐level risk. These findings emphasize the importance of integrated surveillance approaches that consider community‐level factors when guiding outbreak preparedness in similar population settings.

## Author Contributions

**Nushrat Nazia:** conceptualization, methodology, formal analysis, writing – original draft, writing – review and editing. **Mark Loeb:** conceptualization, funding acquisition, supervision, writing – review and editing. **Eleanor Pullenayegum:** methodology, statistical guidance, validation, writing – review and editing. All authors have read and approved the final manuscript.

## Funding

This study was supported by grants from the Canadian Institutes of Health Research (CIHR).

## Ethics Statement

This study was approved by the McMaster University Research Ethics Board (REB #XXXX). All participants provided informed consent as part of the original randomized influenza vaccine trial.

## Conflicts of Interest

M.L. participated in vaccine advisory boards for Pfizer, Seqirus, Sanofi, Merck, Aramis, and GSK.

## Supporting information


**Table S1:** Model specifications and fit statistics for Influenza A/B (all Influenza) outcome.
**Table S2:** Model specifications and fit statistics for Influenza A outcome.
**Table S3:** Model specifications and fit statistics for Influenza B outcome.
**Table S4:** Hyperparameter estimates from Bayesian hierarchical models of influenza outcomes.
**Table S5:** Post hoc fixed‐effect estimates from post hoc multilevel models of the three influenza outcomes.
**Figure S1:** Residual plot showing linearity of temperature effect (Influenza A/B).
**Figure S2:** Residual plot showing linearity of precipitation effect (Influenza A/B).

## Data Availability

Individual‐level data from the Hutterite influenza trial were obtained under institutional and ethical agreements with McMaster University and are not publicly available to protect participant confidentiality. Deidentified data may be available from the corresponding author (M.L.) upon reasonable request and with appropriate approvals. Environmental and geographic variables were derived from publicly available sources, including the Government of Canada Open Data portal and Environment and Climate Change Canada's Historical Climate Data platform. These datasets can be freely accessed through their respective repositories. The analytic code used for statistical modeling is available from the corresponding author upon reasonable request.

## References

[1] C. S. B. Tyrrell , J. L. Y. Allen , and E. Gkrania‐Klotsas , “Influenza: Epidemiology and Hospital Management,” Medicine (Baltimore) 49 (2021): 797–804. 10.1016/j.mpmed.2021.09.015 PMC8624711 34849086

[2] Influenza (Seasonal). Available at https://www.who.int/news‐room/fact‐sheets/detail/influenza‐(seasonal). Accessed 16 September 2025.

[3] E. A. Belongia , M. D. Simpson , J. P. King , et al., “Variable Influenza Vaccine Effectiveness by Subtype: A Systematic Review and Meta‐Analysis of Test‐Negative Design Studies,” Lancet Infectious Diseases 16 (2016): 942–951. 27061888 10.1016/S1473-3099(16)00129-8

[4] L. A. Grohskopf , J. M. Ferdinands , L. H. Blanton , K. R. Broder , and J. Loehr , “Prevention and Control of Seasonal Influenza With Vaccines: Recommendations of the Advisory Committee on Immunization Practices—United States, 2024‐25 Influenza Season,” MMWR ‐ Recommendations and Reports 73 (2024): 1–25. 10.15585/mmwr.rr7305a1 PMC11501009 39197095

[5] M. Darvishian , F. Dijkstra , E. van Doorn , et al., “Influenza Vaccine Effectiveness in the Netherlands From 2003/2004 through 2013/2014: The Importance of Circulating Influenza Virus Types and Subtypes,” PLoS ONE 12 (2017): e0169528. 28068386 10.1371/journal.pone.0169528 PMC5222508

[6] A. M. Near , J. Tse , Y. Young‐Xu , D. K. Hong , and C. M. Reyes , “Burden of Influenza Hospitalization Among High‐risk Groups in the United States,” BMC Health Services Research 22 (2022): 1209. 36171601 10.1186/s12913-022-08586-y PMC9520810

[7] M. Moore , E. Chan , N. Lurie , A. G. Schaefer , D. M. Varda , and J. A. Zambrano , “Strategies to Improve Global Influenza Surveillance: A Decision Tool for Policymakers,” BMC Public Health 8 (2008): 186. 18507852 10.1186/1471-2458-8-186 PMC2430963

[8] K. E. Lafond , R. M. Porter , M. J. Whaley , et al., “Global Burden of Influenza‐Associated Lower Respiratory Tract Infections and Hospitalizations Among Adults: A Systematic Review and Meta‐Analysis,” PLoS Medicine 18 (2021): e1003550. 33647033 10.1371/journal.pmed.1003550 PMC7959367

[9] S. A. Hudu , A. O. Jimoh , A. Alqtaitat , and F. E‐lmigdadi , “The Role of Seasonal Influenza in Compounding the Outbreak of Infectious Diseases: A Critical Review,” Biomedical and Pharmacology Journal 17 (2024): 1–13.

[10] T. M. Quandelacy , C. Viboud , V. Charu , M. Lipsitch , and E. Goldstein , “Age‐ and Sex‐related Risk Factors for Influenza‐Associated Mortality in the United States Between 1997–2007,” American Journal of Epidemiology 179 (2014): 156–167. 24190951 10.1093/aje/kwt235 PMC3873104

[11] B. L. Coleman , S. A. Fadel , T. Fitzpatrick , and S.‐M. Thomas , “Risk Factors for Serious Outcomes Associated With Influenza Illness in High‐ Versus Low‐ and Middle‐Income Countries: Systematic Literature Review and Meta‐Analysis,” Influenza and Other Respiratory Viruses 12 (2018): 22–29. 29197154 10.1111/irv.12504 PMC5818335

[12] Full Article: The Burden of Influenza Complications in Different High‐risk Groups: A Targeted Literature Review. Available at https://www.tandfonline.com/doi/full/10.3111/13696998.2012.752376. Accessed 16 September 2025. 10.3111/13696998.2012.752376 23173567

[13] M. Chittaganpitch , S. Waicharoen , T. Yingyong , et al., “Viral Etiologies of Influenza‐Like Illness and Severe Acute Respiratory Infections in Thailand,” Influenza and Other Respiratory Viruses 12 (2018): 482–489. 29518269 10.1111/irv.12554 PMC6005612

[14] J. Paireau , C. Pelat , C. Caserio‐Schönemann , et al., “Mapping Influenza Activity in Emergency Departments in France Using Bayesian Model‐Based Geostatistics,” Influenza and Other Respiratory Viruses 12 (2018): 772–779. 30055089 10.1111/irv.12599 PMC6185885

[15] J. D. Tamerius , J. Shaman , W. J. Alonso , et al., “Environmental Predictors of Seasonal Influenza Epidemics Across Temperate and Tropical Climates,” PLoS Pathogens 9 (2013): e1003194. 23505366 10.1371/journal.ppat.1003194 PMC3591336

[16] A. V. Diez Roux and A. E. Aiello , “Multilevel Analysis of Infectious Diseases,” Journal of Infectious Diseases 191 (2005): S25–S33. 15627228 10.1086/425288

[17] A. V. Diez‐Roux , “Multilevel Analysis in Public Health Research,” Annual Review of Public Health 21 (2000): 171–192. 10.1146/annurev.publhealth.21.1.171 10884951

[18] R. F. Dedrick , J. M. Ferron , M. R. Hess , et al., “Multilevel Modeling: A Review of Methodological Issues and Applications,” Review of Educational Research 79 (2009): 69–102.

[19] F. Sera and P. Ferrari , “A Multilevel Model to Estimate the Within‐ and the Between‐Center Components of the Exposure/Disease Association in the EPIC Study,” PLoS ONE 10 (2015): e0117815. 25785729 10.1371/journal.pone.0117815 PMC4365026

[20] M. Loeb , M. L. Russell , L. Moss , et al., “Effect of Influenza Vaccination of Children on Infection Rates in Hutterite Communities: A Randomized Trial,” JAMA 303 (2010): 943–950. 20215608 10.1001/jama.2010.250

[21] B. Wang , M. L. Russell , L. Moss , et al., “Effect of Influenza Vaccination of Children on Infection Rate in Hutterite Communities: Follow‐Up Study of a Randomized Trial,” PLoS ONE 11 (2016): e0167281. 27977707 10.1371/journal.pone.0167281 PMC5157992

[22] C. E. McCulloch , S. R. Searle , and J. M. Neuhaus , Generalized, Linear, and Mixed Models, 2nd ed. (Wiley, 2008).

[23] H. Rue , S. Martino , and N. Chopin , “Approximate Bayesian Inference for Latent Gaussian Models by Using Integrated Nested Laplace Approximations,” Journal of the Royal Statistical Society, Series B: Statistical Methodology 71 (2009): 319–392.

[24] X. Wang , Y. R. Yue , and J. J. Faraway , Bayesian Regression Modeling With INLA (Chapman and Hall/CRC, 2018), 324.

[25] L. Held , M. Höhle , and M. Hofmann , “A Statistical Framework for the Analysis of Multivariate Infectious Disease Surveillance Counts,” Statistical Modelling 5 (2005): 187–199.

[26] C. Imai and M. Hashizume , “A Systematic Review of Methodology: Time Series Regression Analysis for Environmental Factors and Infectious Diseases,” Tropical Medicine and Health 43 (2015): 1–9. 10.2149/tmh.2014-21 PMC4361341 25859149

[27] Z. J. Madewell , L.‐P. Wang , N. E. Dean , et al., “Interactions Among Acute Respiratory Viruses in Beijing, Chongqing, Guangzhou, and Shanghai, China, 2009–2019,” Influenza and Other Respiratory Viruses 17 (2023): e13212. 37964991 10.1111/irv.13212 PMC10640964

[28] G.‐A. Fuglstad , D. Simpson , F. Lindgren , and H. Rue , “Constructing Priors That Penalize the Complexity of Gaussian Random Fields,” Journal of the American Statistical Association 114 (2019): 445–452.

[29] D. Simpson , H. Rue , A. Riebler , T. G. Martins , and S. H. Sørbye , “Penalising Model Component Complexity: A Principled, Practical Approach to Constructing Priors,” Statistical Science 32 (2017): 1–28.

[30] A. Riebler , S. H. Sørbye , D. Simpson , and H. Rue , “An Intuitive Bayesian Spatial Model for Disease Mapping That Accounts for Scaling,” Statistical Methods in Medical Research 25 (2016): 1145–1165. 27566770 10.1177/0962280216660421

[31] D. P. Simpson , H. Rue , T. G. Martins , A. Riebler and S. H. Sørbye . “Penalising Model Component Complexity: A Principled, Practical Approach to Constructing Priors,” 2015 August.

[32] A. Peci , A.‐L. Winter , Y. Li , et al., “Effects of Absolute Humidity, Relative Humidity, Temperature, and Wind Speed on Influenza Activity in Toronto, Ontario, Canada,” Applied and Environmental Microbiology 85 (2019): e02426–e02418. 30610079 10.1128/AEM.02426-18 PMC6414376

[33] K. Dave and P. C. Lee , “Global Geographical and Temporal Patterns of Seasonal Influenza and Associated Climatic Factors,” Epidemiologic Reviews 41 (2019): 51–68. 31565734 10.1093/epirev/mxz008

[34] L. Goodwins , B. Menzies , N. Osborne , and D. Muscatello , “Human Seasonal Influenza and Climate Change: A Systematic Review of the Methods Used to Examine the Relationship Between Meteorological Variables and Influenza,” Environmental Epidemiology 3 (2019): 137.

[35] C. Viboud , P.‐Y. Boëlle , F. Carrat , A.‐J. Valleron , and A. Flahault , “Prediction of the Spread of Influenza Epidemics by the Method of Analogues,” American Journal of Epidemiology 158 (2003): 996–1006. 14607808 10.1093/aje/kwg239

