# Supplementary material for: Individual and Environmental Factors Influencing Influenza Transmission: A Multilevel Analysis
Source: Influenza Other Respir Viruses. 2026 Feb 4;20(2):e70232. doi: 10.1111/irv.70232 (PMC12869838; doi:10.1111/irv.70232)

**Supplementary Materials**

**Appendix A**

**Table S1: Model specifications and fit statistics for Influenza A/B (all Influenza) outcome**

| Model No. | Model Name | Description | Weather Terms | WAIC | DIC | LPML |
| --- | --- | --- | --- | --- | --- | --- |
| 1 | **Baseline model (lag-0 weather)** | Includes all individual- and colony-level covariates; current-week mean temperature and precipitation. | Continuous, lag-0 | 2526.90 | 2539.28 | -1263.46 |
| 2 | **Excluding zero-case colonies** | Same as Model 1, but removes colonies with no influenza cases over the study period. | Continuous, lag-0 | 2497.68 | 2501.69 | -1248.85 |
| 3 | **Without vaccination covariates** | Same as Model 1, but excludes individual vaccination status and study group assignment. | Continuous, lag-0 | 2525.50 | 2538.52 | -1262.76 |
| 4 | **Temperature-only model** | Uses only temperature as a predictor; excludes precipitation and vaccination covariates. | Continuous, lag-0 | 2596.68 | 2608.08 | -1298.35 |
| 5 | **Lagged weather model (primary)** | Includes all covariates; uses mean temperature and precipitation lagged by one week. | Continuous, lag-1 | 2526.04 | 2538.59 | -1263.03 |
| 6 | **Categorical weather model** | Uses categorized temperature and precipitation (lagged by one week). | Categorical, lag-1 | 2493.54 | 2505.93 | -1246.78 |
| 7 | **Post-Hoc Analysis (Lagged weather + prior cases)** | Adds binary indicator of influenza cases in previous week; adjusts for full set of covariates. | Continuous, lag-1 + prior week | 2274.76 | 2266.03 | -1227.45 |
| 8 | **Post-Hoc Analysis (Categorical weather + prior cases)** | Same as Model 7, but with temperature and precipitation entered as categorical terms. | Categorical, lag-1 + prior week | 2260.08 | 2249.43 | -1228.00 |

***DIC****:Deviance Information Criterion ;* ***WAIC****: Widely Applicable Information Criterion* *;* ***LMPL****:*Log Marginal Pseudo Likelihood

**Table S2: Model specifications and fit statistics for Influenza A outcome**

| Model No. | Model Name | Description | Weather Terms | WAIC | DIC | LPML |
| --- | --- | --- | --- | --- | --- | --- |
| 1 | **Baseline model (lag-0 weather)** | Includes all individual- and colony-level covariates; current-week mean temperature and precipitation. | Continuous, lag-0 | 1405.07 | 1417.11 | -702.91 |
| 2 | **Excluding zero-case colonies** | Same as Model 1, but removes colonies with no influenza cases over the study period. | Continuous, lag-0 | 1368.71 | 1372.81 | -684.36 |
| 3 | **Without vaccination covariates** | Same as Model 1, but excludes individual vaccination status and study group assignment. | Continuous, lag-0 | 1399.89 | 1416.79 | -700.05 |
| 4 | **Temperature-only model** | Uses only temperature as a predictor; excludes precipitation and vaccination covariates. | Continuous, lag-0 | 1398.27 | 1411.05 | -699.14 |
| 5 | **Lagged weather model (primary)** | Includes all covariates; uses mean temperature and precipitation lagged by one week. | Continuous, lag-1 | 1393.79 | 1408.74 | -696.90 |
| 6 | **Categorical weather model** | Uses categorized temperature and precipitation (lagged by one week). | Categorical, lag-1 | 1355.58 | 1371.08 | -677.78 |
| 7 | **Post-Hoc Analysis (Lagged weather + prior cases)** | Adds binary indicator of influenza cases in previous week; adjusts for full set of covariates. | Continuous, lag-1 + prior week | 1578.60 | 1640.28 | -709.05 |
| 8 | **Post-Hoc Analysis (Categorical weather + prior cases)** | Same as Model 7, but with temperature and precipitation entered as categorical terms. | Categorical, lag-1 + prior week | 1248.91 | 1258.87 | -719.00 |

***DIC****:Deviance Information Criterion ;* ***WAIC****: Widely Applicable Information Criterion* *;* ***LMPL****:*Log Marginal Pseudo Likelihood

**Table S3: Model specifications and fit statistics for Influenza B outcome**

| Model No. | Model Name | Description | Weather Terms | WAIC | DIC | LPML |
| --- | --- | --- | --- | --- | --- | --- |
| 1 | **Baseline model (lag-0 weather)** | Includes all individual- and colony-level covariates; current-week mean temperature and precipitation. | Continuous, lag-0 | 1082.63 | 1096.36 | -541.35 |
| 2 | **Excluding zero-case colonies** | Same as Model 1, but removes colonies with no influenza cases over the study period. | Continuous, lag-0 | 1061.37 | 1064.93 | -530.69 |
| 3 | **Without vaccination covariates** | Same as Model 1, but excludes individual vaccination status and study group assignment. | Continuous, lag-0 | 1080.01 | 1093.88 | -540.03 |
| 4 | **Temperature-only model** | Uses only temperature as a predictor; excludes precipitation and vaccination covariates. | Continuous, lag-0 | 1182.74 | 1197.21 | -591.38 |
| 5 | **Lagged weather model (primary)** | Includes all covariates; uses mean temperature and precipitation lagged by one week. | Continuous, lag-1 | 1055.45 | 1072.61 | -527.72 |
| 6 | **Categorical weather model** | Uses categorized temperature and precipitation (lagged by one week). | Categorical, lag-1 | 1051.42 | 1064.48 | -525.70 |
| 7 | **Post-Hoc Analysis (Lagged weather + prior cases)** | Adds binary indicator of influenza cases in previous week; adjusts for full set of covariates. | Continuous, lag-1 + prior week | 968.07 | 979.22 | -582.76 |
| 8 | **Post-Hoc Analysis (Categorical weather + prior cases)** | Same as Model 7, but with temperature and precipitation entered as categorical terms. | Categorical, lag-1 + prior week | 7396.96 | 1846.5 | -580.95 |

***DIC****:Deviance Information Criterion ;* ***WAIC****: Widely Applicable Information Criterion* *;* ***LMPL****:*Log Marginal Pseudo Likelihood

**Table S4: Hyperparameter estimates from Bayesian hierarchical models of influenza outcomes**

| Outcome | Random effect | Mean | SD | 2.5% CrI | Median | 97.5% CrI | Mode |
| --- | --- | --- | --- | --- | --- | --- | --- |
| All influenza | Precision (S1COL) | 0.462 | 0.122 | 0.270 | 0.446 | 0.746 | 0.416 |
|  | Precision (week) | 1.114 | 0.394 | 0.515 | 1.056 | 2.046 | 0.949 |
|  | Rho (week) | 0.687 | 0.107 | 0.443 | 0.700 | 0.858 | 0.726 |
| Influenza A | Precision (S1COL) | 0.367 | 0.097 | 0.208 | 0.356 | 0.589 | 0.337 |
|  | Precision (week) | 1.399 | 0.485 | 0.679 | 1.323 | 2.563 | 1.182 |
|  | Rho (week) | 0.165 | 0.205 | –0.251 | 0.171 | 0.545 | 0.183 |
| Influenza B | Precision (S1COL) | 0.338 | 0.084 | 0.200 | 0.329 | 0.528 | 0.313 |
|  | Precision (week) | 0.377 | 0.151 | 0.167 | 0.349 | 0.751 | 0.299 |
|  | Rho (week) | 0.664 | 0.095 | 0.447 | 0.674 | 0.818 | 0.697 |

***CrI: Control Interval***

**Table S5: Post-hoc Fixed-effect estimates from post-hoc mulit-level models of the three influenza outcomes**

| Covariate | All Influenza (RR, 95% CrI) | Influenza A (RR, 95% CrI) | Influenza B (RR, 95% CrI) |
| --- | --- | --- | --- |
| Sex (female vs male) | 0.74 (0.57–0.98) | 0.73 (0.50–1.06) | 0.73 (0.49–1.08) |
| Age (per year) | 0.97 (0.96–0.98) | 0.97 (0.95–0.99) | 0.93 (0.90–0.95) |
| High-risk status | 0.90 (0.63–1.28) | 1.10 (0.68–1.78) | 0.41 (0.22–0.77) |
| Individual vaccination | 1.31 (0.89–1.92) | 0.95 (0.53–1.83) | 1.13 (0.59–2.17) |
| Study group (intervention) | 0.30 (0.11–0.80) | 0.18 (0.05–0.67) | 0.96 (0.23–4.15) |
| Distance to city (scaled) | 0.66 (0.37–1.13) | 0.72 (0.36–1.39) | 0.71 (0.32–1.53) |
| Elevation (scaled) | 0.77 (0.48–1.21) | 1.12 (0.60–2.08) | 0.64 (0.34–1.21) |
| Cases in colony (previous week) | 23.3 (14.4–37.7) | 45.9 (20.2–111.0) | 8.58 (4.07–18.1) |
| Temperature × Cases | 1.05 (0.98–1.13) | 1.03 (0.95–1.12) | 1.22 (1.04–1.44) |
| Temperature × No Cases | 0.99 (0.95–1.04) | 0.91 (0.88–0.95) | 1.15 (1.06–1.27) |
| Precipitation × Cases | 1.16 (0.69–1.95) | 1.52 (0.76–3.07) | 1.19 (0.49–2.86) |

**Appendix B**

**Figure S1: Residual plot showing linearity of temperature effect (Influenza A/B)**

**
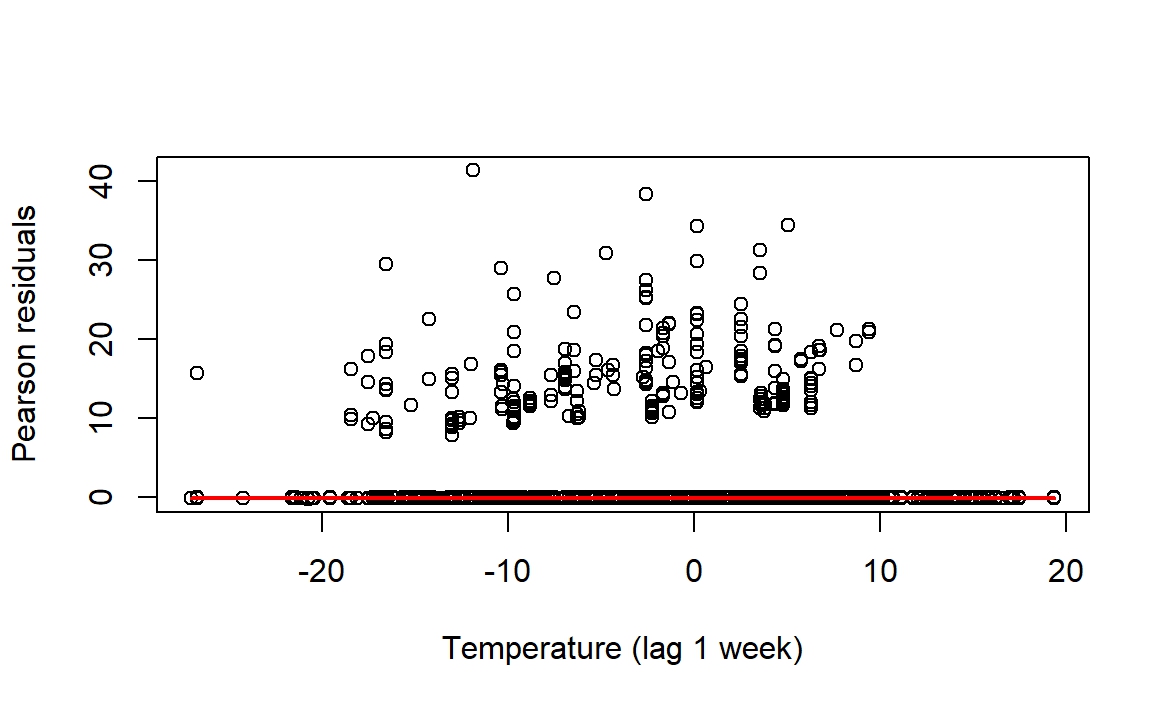
**

**Figure S2: Residual plot showing linearity of precipitation effect (Influenza A/B)**


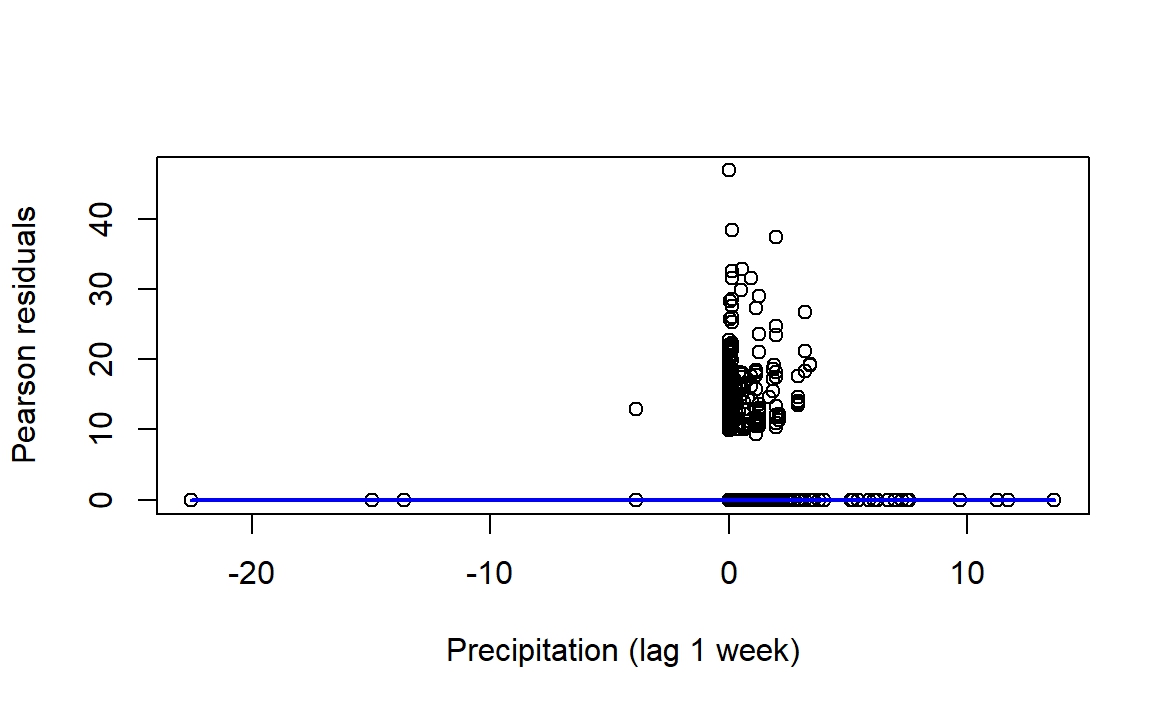

Supplement: Supplementary file 1 — Table S1: Model specifications and fit statistics for Influenza A/B (all Influenza) outcome. Table S2: Model specifications and fit statistics for Influenza A outcome. Table S3: Model specifications and fit statistics for Influenza B outcome. Table S4: Hyperparameter estimates from Bayesian hierarchical models of influenza outcomes. Table S5: Post hoc fixed‐effect estimates from post hoc multilevel models of the three influenza outcomes. Figure S1: Residual plot showing linearity of temperature effect (Influenza A/B). Figure S2: Residual plot showing linearity of precipitation effect (Influenza A/B). [file IRV-20-e70232-s001.docx]
